# Inhibition by Somatostatin Interneurons in Olfactory Cortex

**DOI:** 10.3389/fncir.2016.00062

**Published:** 2016-08-17

**Authors:** Adam M. Large, Nicholas A. Kunz, Samantha L. Mielo, Anne-Marie M. Oswald

**Affiliations:** Department of Neuroscience and Center for the Neural Basis of Cognition, University of PittsburghPittsburgh, PA, USA

**Keywords:** somatostatin interneurons, parvalbumin interneurons, optogenetic stimulation, piriform cortex, inhibitory connections

## Abstract

Inhibitory circuitry plays an integral role in cortical network activity. The development of transgenic mouse lines targeting unique interneuron classes has significantly advanced our understanding of the functional roles of specific inhibitory circuits in neocortical sensory processing. In contrast, considerably less is known about the circuitry and function of interneuron classes in piriform cortex, a paleocortex responsible for olfactory processing. In this study, we sought to utilize transgenic technology to investigate inhibition mediated by somatostatin (SST) interneurons onto pyramidal cells (PCs), parvalbumin (PV) interneurons, and other interneuron classes. As a first step, we characterized the anatomical distributions and intrinsic properties of SST and PV interneurons in four transgenic lines (SST-cre, GIN, PV-cre, and G42) that are commonly interbred to investigate inhibitory connectivity. Surprisingly, the distributions SST and PV cell subtypes targeted in the GIN and G42 lines were sparse in piriform cortex compared to neocortex. Moreover, two-thirds of interneurons recorded in the SST-cre line had electrophysiological properties similar to fast spiking (FS) interneurons rather than regular (RS) or low threshold spiking (LTS) phenotypes. Nonetheless, like neocortex, we find that SST-cells broadly inhibit a number of unidentified interneuron classes including putatively identified PV cells and surprisingly, other SST cells. We also confirm that SST-cells inhibit pyramidal cell dendrites and thus, influence dendritic integration of afferent and recurrent inputs to the piriform cortex. Altogether, our findings suggest that SST interneurons play an important role in regulating both excitation and the global inhibitory network during olfactory processing.

## Introduction

Throughout the cortex, inhibitory interneurons that express somatostatin (SST) have been implicated in numerous aspects of sensory processing including gain control and/or tuning (Adesnik et al., 2012; Wilson et al., 2012; Stryker, 2014; Seybold et al., 2015; Sturgill and Isaacson, 2015). SST interneurons inhibit excitatory pyramidal cells (PCs; Fino and Yuste, 2011; Pfeffer et al., 2013) as well as inhibitory interneurons, including parvalbumin (PV) cells (Pfeffer et al., 2013; Xu et al., 2013; Jiang et al., 2015). Thus, SST-cells can both directly inhibit and indirectly disinhibit PCs (Cottam et al., 2013; Xu et al., 2013). With respect to direct inhibition, SST-cells primarily inhibit pyramidal cell dendrites and regulate postsynaptic Ca^2+^ signals, spike bursts, spine, and synapse dynamics (Chiu et al., 2013; Marlin and Carter, 2014; Chen et al., 2015). In addition, ongoing SST-cell activity can also modulate excitatory transmission through GABAergic activation of presynaptic GABA_B_ receptors (Urban-Ciecko et al., 2015). In contrast to extensive analysis of SST-mediated inhibition in neocortex, little is known about the circuitry of SST-cells in piriform cortex. In this study, we investigate SST-cell properties and inhibitory connectivity in the piriform cortex to gain insight into the roles these interneurons play in olfactory processing.

The anterior piriform cortex (APC) is a trilaminar paleocortex responsible for processing olfactory information (Wilson and Sullivan, 2011; Bekkers and Suzuki, 2013). PCs are found in layer (L)2/3 and project apical dendrites to L1 to receive direct input from the olfactory bulb (L1A) as well as intracortical excitation (L1B). In addition, PCs project axons throughout L2/3 to recruit feedback or recurrent inhibition from a number of interneuron classes including SST-cells and PV neurons (Haberly and Price, 1978; Suzuki and Bekkers, 2010a,b, 2012). SST-expressing interneurons account for ~30% of interneurons in piriform cortex (Suzuki and Bekkers, 2010b). SST-cells have been shown to extend axons to L1B as well as make synaptic connections with PCs (Suzuki and Bekkers, 2010a,b), although direct dendritic inhibition has not been verified. This suggests that SST-interneurons are poised to regulate dendritic excitability as in neocortical circuits. More recently, optogenetic inactivation of SST-cells revealed that SST-interneuron activity narrows odor-tuning curves through subtractive inhibition (Sturgill and Isaacson, 2015). However, it is unclear whether the mechanism involves direct inhibition of PCs, inhibitory interneurons, or both. These studies highlight the importance of understanding how SST-interneurons participate in APC circuits to affect the activity of both pyramidal cell and inhibitory interneurons.

In this study we characterized the expression patterns, intrinsic physiology, and inhibitory connectivity of SST-interneurons in the APC. We used commercially available transgenic mouse lines that target either SST cells (SST-cre and GIN) or PV-cells (PV-cre and G42). These lines are commonly crossed to express GFP in target postsynaptic cells [GIN, G42, (Oliva et al., 2000; Chattopadhyaya et al., 2007), and channelrhodopsin (ChR2) in presynaptic cells (cre-lines, (Madisen et al., 2012)]. However, we find that the densities and electrophysiological properties of GIN and G42 cells are not representative of the majority of SST and PV interneurons in APC ruling out this strategy. Instead we expressed ChR2 in SST-cre animals and used a clustering algorithm to differentiate postsynaptic interneurons based on intrinsic properties. As expected from neocortical studies, we found that SST-interneurons broadly inhibited a variety of interneuron classes, including putative PV cells. However, in contrast to neocortex (Pfeffer et al., 2013), we also found that SST-cells can strongly inhibit each other. Further, we confirm that SST-cells inhibit the distal dendritic and perisomatic regions of PCs. Finally, we discuss potential limitations of these findings due to the expression strategies utilized. Altogether, we provide new information about the distributions and intrinsic properties of SST-cell subtypes in commonly used transgenic lines as well as the inhibitory connectivity of SST-cells in APC circuits. These findings are first step toward understanding the roles SST-interneurons play in odor processing in piriform cortex.

## Methods

### Mice

We used six commercially available transgenic mouse lines in this study obtained from Jackson Laboratories. GIN (FVB-Tg(GadGFP)45704Swn/J) and G42(CB6-Tg(Gad1-EGFP)G42Zjh/J) mice express green fluorescent protein (GFP) in subsets of SST and PV interneurons, respectively. SST-cre (SST^*tm2.1*(*cre*)*Zjh*^/J) and PV-cre (*Pvalb*^*tm1*(*cre*)*Arbr*^/J) mice express cre-recombinase under the promoters for SST and PV, respectively. We crossed these cre mice with Ai14 (B6;129S6-*Gt(ROSA)26Sor*^*tm14*(*CAG*−*tdTomato*)*Hze*^/J) or Ai32 (B6.Cg-*Gt(ROSA)26Sor*^*tm32*(*CAG*−COP4^*^H134R/*EYFP*)*Hze*^/J) mice to produce offspring that express tdTomato (SST-tdTom, PV-tdTom) or channelrhodopsin (SST-ChR2), respectively. Mice of both sexes were used in all experiments. The University of Pittsburgh IACUC approved all procedures.

### Anatomy

GIN, G42, SST-tdTom, or PV-TdTom mice (P200–300) were given an overdose (500 μl) of ketamine (100 mg/kg) and xylazine (10 mg/kg) cocktail then transcardially perfused with ice cold phosphate buffered saline (PBS) followed by 4% paraformaldehyde (PFA). Brains were removed and post-fixed for 24 h in 4% PFA then sunk in 30% sucrose solution overnight. Coronal sections (50 μm) were cut on a freezing microtome and maintained in phosphate buffer prior to immunochemistry or mounting. Every other section was mounted using fluoromount to protect fluorescence and minimize background. Sections were imaged on a Nikon Eclipse-Ci microscope at 4–20x magnifications. Illumination was provided by a mercury lamp (Nikon Intensilight) and delivered through appropriate filter blocks for GFP (495 nm) and tdTomato (585 nm). Light intensity and exposure duration (100–400 ms) were optimized for the first section in a series using automated software (Nikon Elements), then maintained for ensuing sections. Sections were photographed using a CCD HD color camera (Nikon DsFi2). Cell counts were obtained using automated software (Nikon Elements, see below).

### Immunochemistry

To minimize background fluorescence and fading, GFP(+) GIN and G42 cells were stained using anti-GFP immunochemistry (1°: rabbit anti-GFP, #A11122 Life Technologies, 1:10,000 dilution, 24 h, 20°C; 2°: donkey anti-rabbit Biotin-SP Affinipure #711-065-0152, Jackson Immunoresearch Laboratories, 1:100 dilution, 1 h, 20°C) followed by avidin-biotin-peroxidase reaction (Elite Kit, Vector Laboratories) using 3,3′-diaminobenzidine (DAB). PV expressing cells were immunostained using rabbit anti-parvalbumin (1°: PV27, Swant, 1:1000, 48 h, 4°C; 2°: donkey anti-rabbit Alexa-fluor-488, #A21206 Life Technologies, 1:500, 3 h, 20°C). SST expressing cells were immunostained using rabbit anti-somatostatin (1°: Ab20067 Immunostar, 1:500, 48 h, 4°C; 2°: donkey anti-rabbit Alexa-fluor-488, #A21206 Life Technologies, 1:500, 3 h, 20°C). Prior to staining, tissue was blocked in 10% normal donkey serum (1 h). Specificity controls for the secondary antibodies were performed by excluding the 1° antibody in a small number of sections (*n* = 4). Only faint neuropil fluorescence was visible and no cell bodies were stained.

### *In vitro* slice preparation

Brain slices of APC were prepared from mice aged P18–30. The mice were anesthetized with isoflurane and decapitated. The brain was removed from the skull and immersed in ice cold oxygenated (95% O_2_-5% CO_2_) ACSF (in mM: 125 NaCl, 2.5 KCl, 25 NaHCO_3_, 1.25 NaH_2_PO_4_, 1.0 MgCl_2_, 25 Dextrose, 2.5 CaCl_2_; all chemicals from Sigma, USA unless otherwise stated). Parasagittal slices (300 μm) were made using a vibratome (Leica Biosystems) in ice cold ACSF. The slices were transferred to warm ACSF (37°C) for 30 min and then rested at 20°–22°C for 1 h prior to recording (31°–35°C).

### Electrophysiology

Whole cell, voltage and current clamp recordings were performed using a MultiClamp 700B amplifier (Molecular Devices, Union City, CA). Data were low pass filtered (4 kHz) and digitized at 10 kHz using an ITC-18 (Instrutech) controlled by custom software (Recording Artist, https://bitbucket.org/rgerkin/recording-artist) written in IgorPro (Wavemetrics). Recording pipettes (4–10 MΩ) were pulled from borosilicate glass (1.5 mm, outer diameter) on a Flaming/Brown micropipette puller (Sutter Instruments). The series resistance (< 22 MΩ) was not corrected. The intracellular solution consisted of (in mM) 130 K-gluconate, 5 KCl, 2 MgCl_2_, 4 ATP-Mg, 0.3 GTP, 10 HEPES, and 10 phosphocreatine, 0.05% biocytin. In a subset of PCs, a Cs-gluconate internal solution was used (100 Gluconic Acid, 5 MgCl_2_, 0.2 EGTA, 40 HEPES, 2 ATP-Mg, 0.3 GTP, 0.05% biocytin, titrated to pH 7.2 with 50% Cs-OH). When IPSCs were recorded, 4.5 μM QX-314 was also added to the internal solution. Recordings were obtained from L2/3 PCs as well as interneurons in lower L2 and L3. Neurons were visualized using infrared-differential interference contrast microscopy (IR-DIC, Olympus). PCs were identified using intrinsic properties and post hoc anatomical reconstruction (Neurolucida). In transgenic mice, interneurons were targeted using red (tdTom) or green (GFP) fluorescence. For studies involving ChR2 stimulation, neurons were targeted based on the absence of yellow fluorescent protein (YFP) fluorescence. In the absence of fluorescence, neural identification was based on intrinsic properties. In all neurons, the input resistance (*R*_in_), time constant (τ_m_), and sag due to *I*_h_ current, were assessed in current clamp using a series of hyperpolarizing and depolarizing current steps (−50 to 50 pA, 1 s duration). Input resistance was the slope of a linear fit of the relationship between the change in voltage and current amplitude for steps between (−50 and 50 pA in 10 pA increments). Membrane time constant was determined based on the monoexponential fit of the falling phase of the voltage response to a −50 pA current injection. Sag was measured as the difference in voltage between the onset (first 50 ms) and last 100 ms of a −50 pA step. To assess spike responses a series of depolarizing steps (1 s duration, 0–1000 pA, 100 pA stepsize) was used. Rheobase was taken as the minimum current to elicit spike responses. Spike width was assessed at rheobase and taken as the average full-width at spike half-height. Interspike interval (ISI) analyses (adaptation ratio and CV) were conducted on spike responses to current injection 100 pA above rheobase. Adaptation ratio corresponded to the last ISI divided by the first ISI, while the CV of the ISI was the standard deviation of the ISI divided by the mean ISI.

### Light stimulation

Blue light (λ = 460–488 nm, GFP block, Olympus) for full-field optical stimulation was provided by metal halide lamp (200W, Prior Scientific) passed through the microscope objective (60x, immersion, Olympus). Based on our measurements, the light intensity at the tissue is estimated to be ~3–4 mW. Light pulses were controlled using a mechanical shutter (Sutter Instruments). Light intensity and duration (20 ms) was chosen to reliably evoke at least one spike in SST-cells although multiple spikes were frequently observed. This was advantageous because weak synapses that undergo short-term potentiation are more likely to be observed. Since these are inhibitory neurons and there is no evidence of depolarizing inhibition at threshold membrane potentials, polysynaptic responses are unlikely under these recording conditions.

### Drug application

The GABA_A_ receptor antagonist, Gabazine (GZ, 40 μM in ACSF) was loaded into a regular patch pipette and locally applied using a gentle positive pressure (< 1–5 s duration) from a 1 cc syringe by hand. GZ was applied within 20 μm of the soma of the recorded cell or in L1B directly above the recorded cell or at both locations simultaneously. Pressure was sufficient to minimally distort tissue in a region ~50 μm around the injection site but did not alter recordings in the absence of GZ. Slices were oriented such that bath flow was perpendicular to the somatodendritic axis of the pyramidal cell to minimize diffusion between somatic and dendritic application sites.

### Data analysis

All summary data is presented as the mean ± standard error (*SE*) with the exception of medians and quartiles (Q_1_, Q_3_) where noted in the text.

#### Cell counts

Neural densities were quantified as number of cells per mm^2^ in regions of interest (ROI) within the ventral anterior piriform cortex (vAPC). Every other coronal section was analyzed spanning 600–1000 μm along the rostral-caudal extent of the APC. For each mouse, densities were averaged across 6–10 sections. To obtain total cell density, ROIs extended from L1A to the anterior commissure encompassing L1B, L2, L3, and when present, endopiriform (EP) areas. For laminar densities, ROIs corresponded to the entirety of each layer within vAPC. In sections from both SST-tdTom and PV-tdTom mice, the neuropil was sufficiently fluorescent to visualize L2 as densely packed dark voids corresponding to unlabeled neurons. These dark voids were more diffuse in L3. Endopiriform areas were difficult to distinguish. In SST-tdTom mice, the L3/EP edge was defined by the transition from larger multipolar cells to smaller elongated tdTom(+) somas (Kowianski et al., 2004). This edge could not be discerned in PV-tdTom mice so counts in EP were not explicitly performed and L3 counts include EP. Counts were made in a single focus plane for each section at 4x magnification chosen to maximize the number of cells in focus (but see exception below). For fluorescent markers (tdTom or GFP) automated cell counts within a region of interest (ROI) were obtained based on fluorescence intensity and circularity using Elements Software (Nikon). Neurons that were immunostained and/or double-labeled (tdTom+GFP) cells were counted by hand within defined ROIs. Compared to PV immunostaining, SST immunostaining was weaker, punctate, and did not always fill the soma. For this reason, cell counts were performed at 20x magnification across a 20–30 μm *z*-stack (1 μm intervals, 2–3 ROIs per section). Two researchers independently verified all counts.

#### Cluster analysis

Neurons were grouped on the basis of subthreshold and/or suprathreshold intrinsic properties using hierarchical clustering algorithm using Ward's method implemented in R [Free Statistics Software, Wessa.net (Wessa, 2012)]. Prior to clustering, all data was standardized to obtain *z*-values. For sequential clustering, defined groups of neurons were removed to enhance differentiation of remaining clusters. In these cases, *z*-values were recalculated using the remaining data set.

#### Analysis of inhibition

Electrophysiology traces of IPSCs are presented as the average across trials for individual neurons. IPSC strength was taken as the area (pAs) under the IPSC. Average IPSCs with minimum amplitude of 10 pA were included for analyses; smaller IPSCs were not distinguishable from noise.

#### Statistics

Due to the nature of the data we used a number of statistical tests. Here, we provide a justification for cases in which the conditions for Student's *t*-test or ANOVA are not met. For small sample sizes (< 10) non-parametric Mann-Whitney *U*-tests (MWU) and Wilcoxon Signed Ranks tests (WSR) were used for unpaired and paired data, respectively. For equal variances and multiple comparisons we used ANOVA with *post-hoc* Tukey Test (ANOVA-Tukey). For multiple comparisons with unequal variance we used Welch's ANOVA (ANOVA-Welch). For groups with unequal variance and sample sizes, multiple comparisons of the distributions were made using the non-parametric, Kruskal-Wallis test (KW-test). The remaining statistical comparisons were made using parametric paired or unpaired Student's *t*-tests or ANOVA without correction. All statistical tests are indicated in the main text.

## Results

We used transgenic mouse lines to target either SST interneurons (GIN and SST-cre) or PV interneurons (G42 or PV-cre). Our initial goal was to cross SST-cre mice with G42 mice to investigate the inhibitory connectivity between SST and PV interneurons. However, these transgenic lines have not been previously characterized in piriform cortex, so we first investigated the anatomical distributions and electrophysiological properties of interneurons targeted in these lines. SST-cre or PV-cre lines were crossed with Ai14 mice to express the red fluorescent protein, tdTomato, in SST and PV-cells (denoted: SST-tdTom and PV-tdTom). The GIN and G42 lines express GFP in SST and PV cells, respectively. To minimize background fluorescence and fading we used anti-GFP immunochemistry to stain GFP(+) cells in tissue from GIN and G42 mice (see Methods).

### Distribution of SST and PV interneurons in piriform cortex

**Figure 1:**
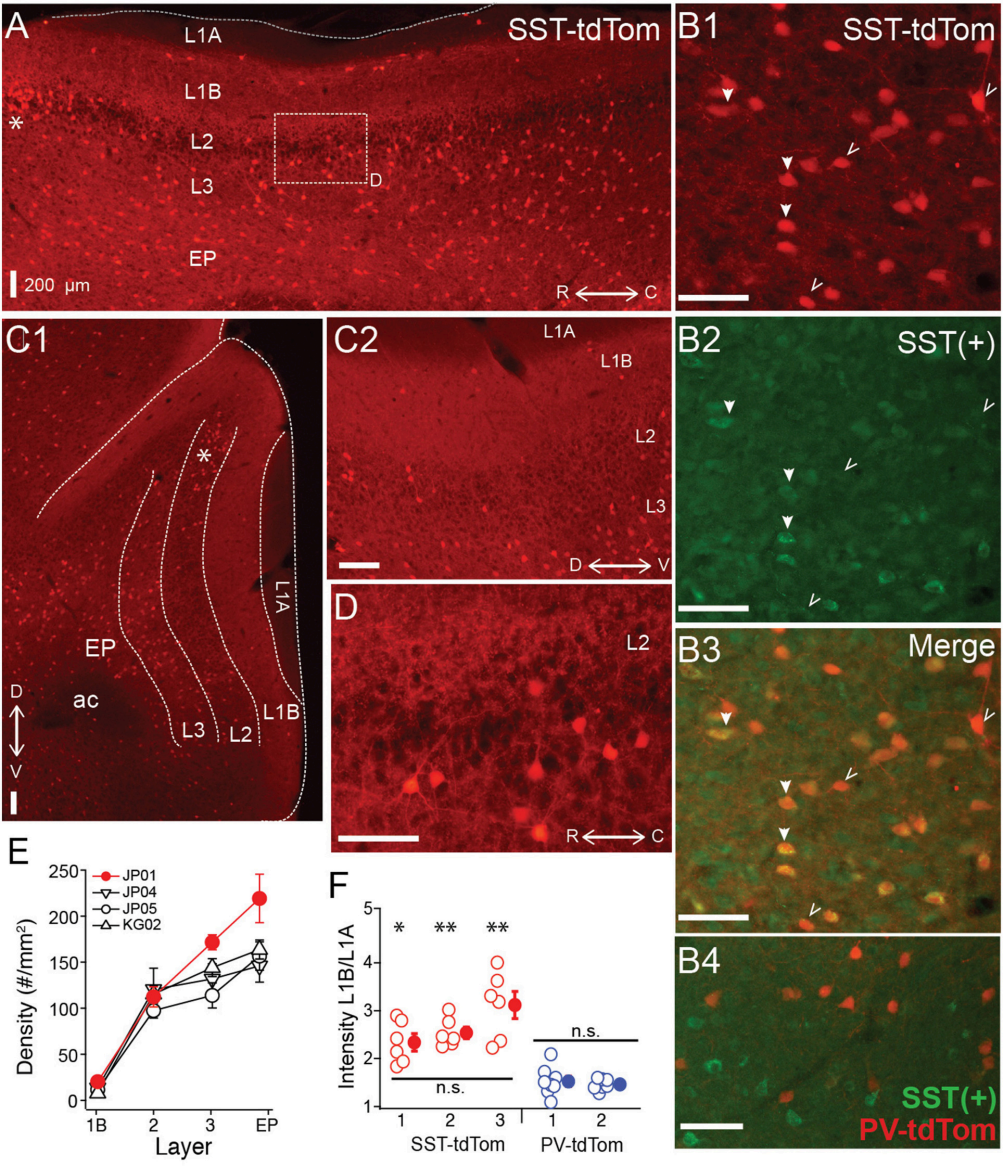
**Distributions of SST-cre cells in anterior piriform cortex. (A)** Sagittal section of anterior piriform cortex from an SST-tdTom mouse (mouse #JP01). Lamina are labeled (L1A, L1B, L2, L3, EP), asterisk (^*^) corresponds to dorsal/rostral L2 density of SST-cre cells. 4x magnification, Scale bar: 200 μm. **(B)** Co-expression of SST-tdTom cells with anti-somatostatin immunolabeling [SST(+), green]. SST(+) cells are shown with filled arrows while SST(−) cells are labeled with open arrows (20x, scale bars 100 μm; **B1**) SST-tdTom expression (red; **B2**) SST-immunolabeling (green; **B3**) Merge of **(B1,B2)** showing co-expression (yellow). **(B4)** Minimal overlap of PV-tdTom (red, mouse KM04) and SST(+) cells (green). **(C1)** Coronal section from opposite hemisphere of mouse #JP01 shown in **(A)**. Labels as in **(A)**, 4x magnification, Scale bar: 200 μm. **(C2)** Enlarged (10x) area of **(C1)** showing tdTom fluorescence in L1B corresponding to projections from SST-cells. Scale bar: 200 μm. **(D)** Enlarged (20x) area of box shown in **(A)**. Scale bar: 100 μm. **(E)** Average density of SST-cells in each layer for four different mice from two different litters (denoted JP, KG). Points shown in red correspond to data from mouse #JP01 shown in panels **(A)** and **(C1,C2)**. **(F)** Ratio of fluorescence in L1B:L1A for three SST-tdTom mice and two PV-tdTom mice. Each point corresponds to one coronal section. The fluorescence ratio did not significantly differ between SST-tdTom mice but was significantly higher in individual SST-tdTom vs. PV-tdTom mice (^*^p < 0.05; ^**^p < 0.01, ANOVA-Welch). ac, anterior commissure.

First we investigated the total density (across layers) as well as laminar densities of tdTom(+) cells in SST-tdTom mice. We focused on the region of vAPC that contains the lateral olfactory tract (L1A). As expected based on immunohistochemistry studies (Suzuki and Bekkers, 2010b), we found a high density (100 ± 5.83 cells/mm^2^, *n* = 6 mice) of SST-tdTom cells in APC (Figure 1A). Co-labeling with an antibody to SST revealed that the majority, 75 ± 3% of SST-tdTom cells express somatostatin [SST(+)] consistent with previous findings (Nassar et al., 2015; Figure 1B, *n* = 7 ROIs, from two mice see Methods). It should be noted that SST immunolabeling varied in intensity across cells and may underestimate true co-labeling. The highest densities of SST-tdTom (*n* = 4 mice, Figures 1A,C1,E) and SST(+) co-labeled cells (84 ± 2%) were found in L3 and endopiriform (EP). A number of other patterns were also apparent. First, a small number of SST-tdTom cells lined the border between L1A and L1B (Figure 1A). Second, with the exception of a cluster of cells at the dorsal edge of the LOT near the rhinal fissure (asterisks, Figures 1A,C1), the density of SST-tdTom cells in L2 was relatively low. Further, the percentage of SST(+) co-labeled cells was lowest in L2 (41 ± 5%). And finally, there was strongly fluorescent neuropil in L1B and L2/3 consistent with dendritic and/or axonal and terminal projections from SST-tdTom cells (Figures 1C2,D). It has been suggested that SST-cells primarily inhibit dendrites while PV-cells inhibit somas. Since L1B is the location of the proximal apical dendrites of PCs, we compared the intensity of this fluorescence between SST-tdTom and PV-tdTom mice. To control for variations in fluorescence across sections and animals, average intensity over a small area (140 μm^2^) in L1B was normalized by the average intensity of a comparable area in the L1A. Only tissue from animals with low background fluorescence in L1A was used (SST-tdTom: *n* = 3 mice, PV-tdTom: *n* = 2 mice). Normalized intensity values (6–8 sections per animal) were compared between all animals. We found that L1B fluorescence did not significantly differ within SST-tdTom or PV-tdTom groups but was significantly higher in SST-tdTom mice compared to PV-tdTom mice (^*^p < 0.05, ^**^p < 0.01; ANOVA-Welch Figure 1F) These findings suggest SST-cells are poised to mediate dendritic inhibition of PCs in L1B.

**Figure 2:**
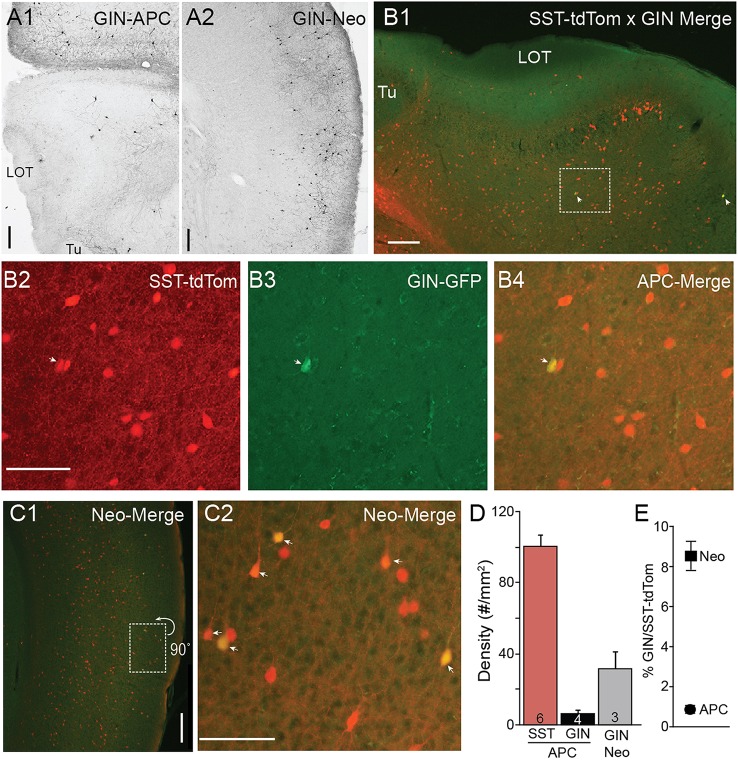
**Minimal co-expression of GFP and tdTom in GIN and SST-cre lines. (A)** GFP(+) GIN cells labeled using anti-GFP antibodies and DAB (see Methods) in sections of anterior piriform cortex **(A1)** and neocortex (Neo) **(A2)** from a GIN mouse. 4x magnification, Scale bar: 200 μm. **(B)** Merged tdTom (SST-cre) and GFP (GIN) fluorescence in APC from an SST-tdTom-GIN mouse. White arrows indicate co-labeled cells. **(B1)** 4x magnification, Scale bar: 200 μm. **(B2–B4)** Region shown in **(B1)** enlarged (20x, scale bar 100 μm) in for SST-tdtom cells (red, **B2**), GIN cells (GFP, **B3**) and merged **(B4)** to show double labeled cells (yellow). Very few SST-tdtom cells double label with GIN cells in APC. **(C1)** Merged tdTom (SST-cre) and GFP (GIN) fluorescence in neocortex from the same SST-tdTom-GIN mouse as in B. **(C2)** Region shown in **(C1)**, enlarged (20x, scale bar 100 μm) showing a higher proportion of SST-tdTom cells co-label with GFP(+) GIN cells (yellow, arrows) in neocortex. Scale bars as in **(B)**. **(D)** Summary plots showing the average density of SST-tdTom cells in APC compared to GIN cells in APC and Neocortex. **(E)** Percent overlap between SST-tdTom and GIN cells in APC and neocortex. Tu, olfactory tubercle.

In neocortex, GIN mice express GFP in a few subtypes of SST-cells including dendrite targeting Martinotti cells in superficial L2/3 (Ma et al., 2006; McGarry et al., 2010). When we analyzed the distributions of GIN cells in piriform cortex (*n* = 4 mice) we found an exceptionally low density of GFP(+) GIN cells (6.21 ± 2.23 cells/mm^2^) compared to SST-tdTom cells in piriform cortex (100 ± 5.83 cells/mm^2^) or GIN cell density in neocortical somatosensory cortex (31.4 ± 9.51 cells/mm^2^, Figures 2A,D). We crossed a heterozygous SST-tdTom mouse with a GIN mouse to quantify double labeling between SST-tdTom and GIN cells. While GIN cells co-localized with SST-tdTom cells, double labeling was sparse in APC, typically only 1–4 cells per section or 1% of SST-tdTom cells (*n* = 2 mice, Figures 2B,E). Double labeling was much higher in neocortex (9%; Figures 2C, E). These overlap percentages were lower than expected based on raw counts of GIN and SST-tdTom cells in APC (~6%) or previous reports in neocortex [15–20%, (Ma et al., 2006)]. The most likely explanation is that the raw GFP fluorescence in GIN cells was weak and difficult to distinguish from background compared to DAB stained tissue. Nonetheless, these findings suggest that SST-cells labeled in the GIN line (i.e., Martinotti cells) are only a very small subset of all SST-cells in APC.

**Figure 3:**
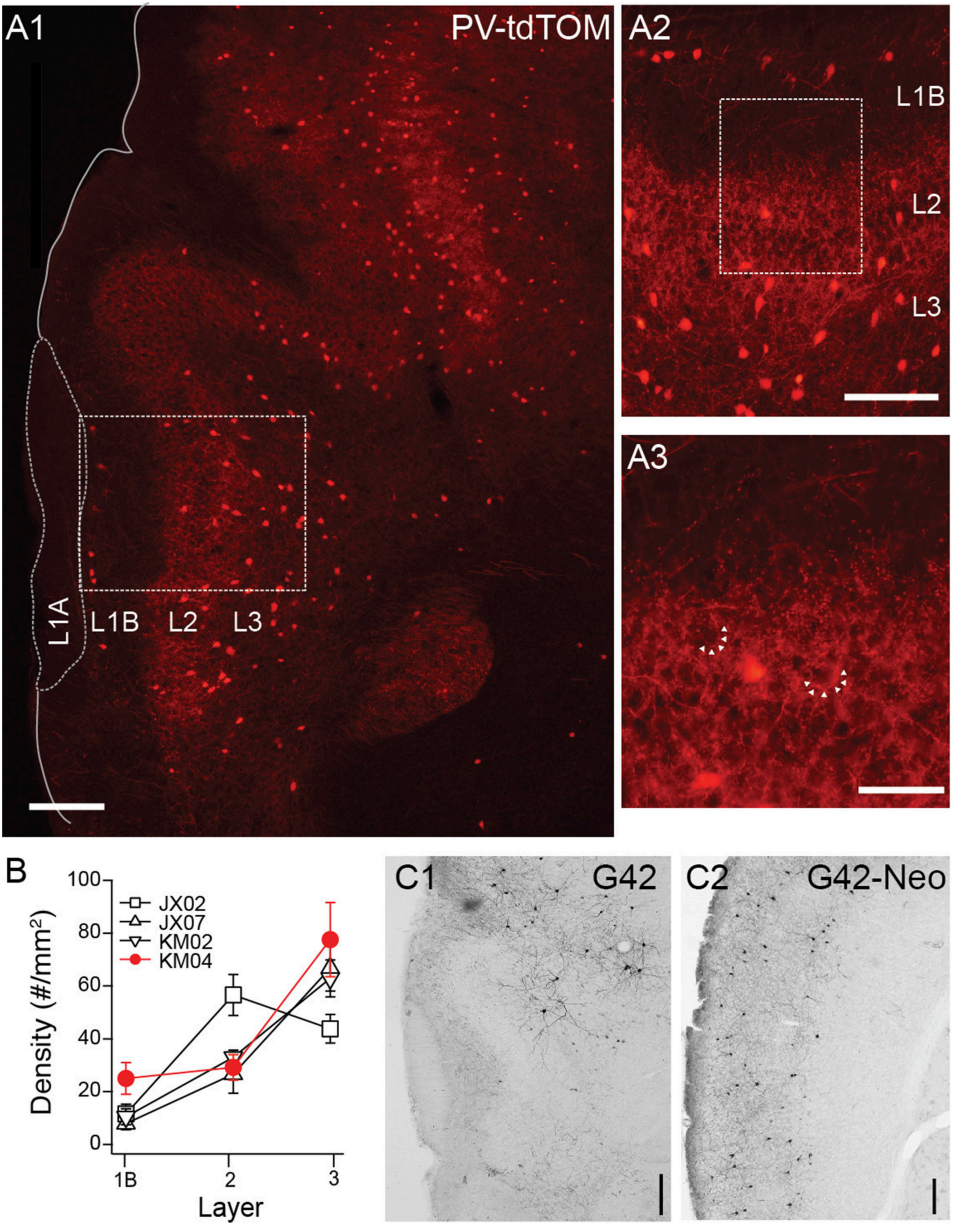
**Distributions of PV-cre cells in anterior piriform cortex. (A1)** Coronal section from a PV-tdTom mouse (KM04). 4x magnification, Scale bar: 200 μm. **(A2)** Enlarged (10x) area of A1 showing a lack tdTom fluorescence in L1B but strong fluorescence in L2. Scale bar: 100 μm. **(A3)** Enlarged (20x) area of box shown in **(A2)**. Note punctate tdTom fluorescence outlining dark voids (arrows) suggestive of basket synapses. Scale bar: 50 μm. **(B)** Average density of PV-cells in each layer for four different mice from two different litters (denoted JX, KM). Points shown in red correspond to data from mouse KM04 shown in panels **(A1–A3)**. **(C)** Anti-GFP labeling in coronal sections from a G42 mouse **(C1)** APC **(C2)** Neocortex (Neo). 4x magnification, Scale bar: 200 μm.

Next we turned our attention to transgenic lines targeting PV interneurons. In PV-tdTom mice (*n* = 5) the densities and laminar distributions of tdTom(+) cells were also consistent with previous reports (Gavrilovici et al., 2010; Suzuki and Bekkers, 2010b). Overall, the average density of PV-tdTom (48.2 ± 2.96 cells/mm^2^, *n* = 5 mice) cells was significantly lower than SST-tdTom cells (100 ± 5.83 cells/mm^2^, *n* = 6 mice p:0.008, MWU-test). The majority of PV-tdTom somas were found at the L2/3 border and extended to deep L3. A small number of tdTom(+) cells were also consistently found at the L1A/B border (Figures 3A,B, *n* = 4 mice). Diffuse fluorescence corresponding to axons and dendrites was most prominent L2/3 (Figures 3A1,A2). Within L2/3, punctate labeling was visible surrounding darker voids (unlabeled somas) consistent with basket-like synapses (Figure 3A3). Finally, PV-tdTom cells sent fewer axonal projections to L1B than SST-cells (Figures 1D, 3A1–A3).

Surprisingly, GFP(+) neurons in the G42 line differed in nearly every aspect from cells in the PV-cre line. First, the density of GFP(+) G42 cells was very low in piriform cortex (11.34 cells/mm^2^, *n* = 1 mouse, Figure 3C1) compared to PV-tdTom cells (48.2 ± 2.96 cells/mm^2^, *n* = 5 mice, Figure 3A) or G42 cells in neocortex (35.67 cells/mm^2^, Figure 3C2). Second, the majority of G42 cells were located deep in L3 or endopiriform areas. This was true both in histological sections and for GFP(+) neurons recorded from G42 mice (data not shown). Since G42 mice are heterozygous, there is low probability of obtaining a triple transgenic mouse (~ < 1/litter) from crosses of G42 and PV-tdTom mice. Thus, we were unable ascertain the overlap between G42 cells and PV-tdTom populations. However, given the sparseness and location of G42 cells we expect that G42 cells represent a very small subpopulation of PV cells in piriform cortex.

**Figure 4:**
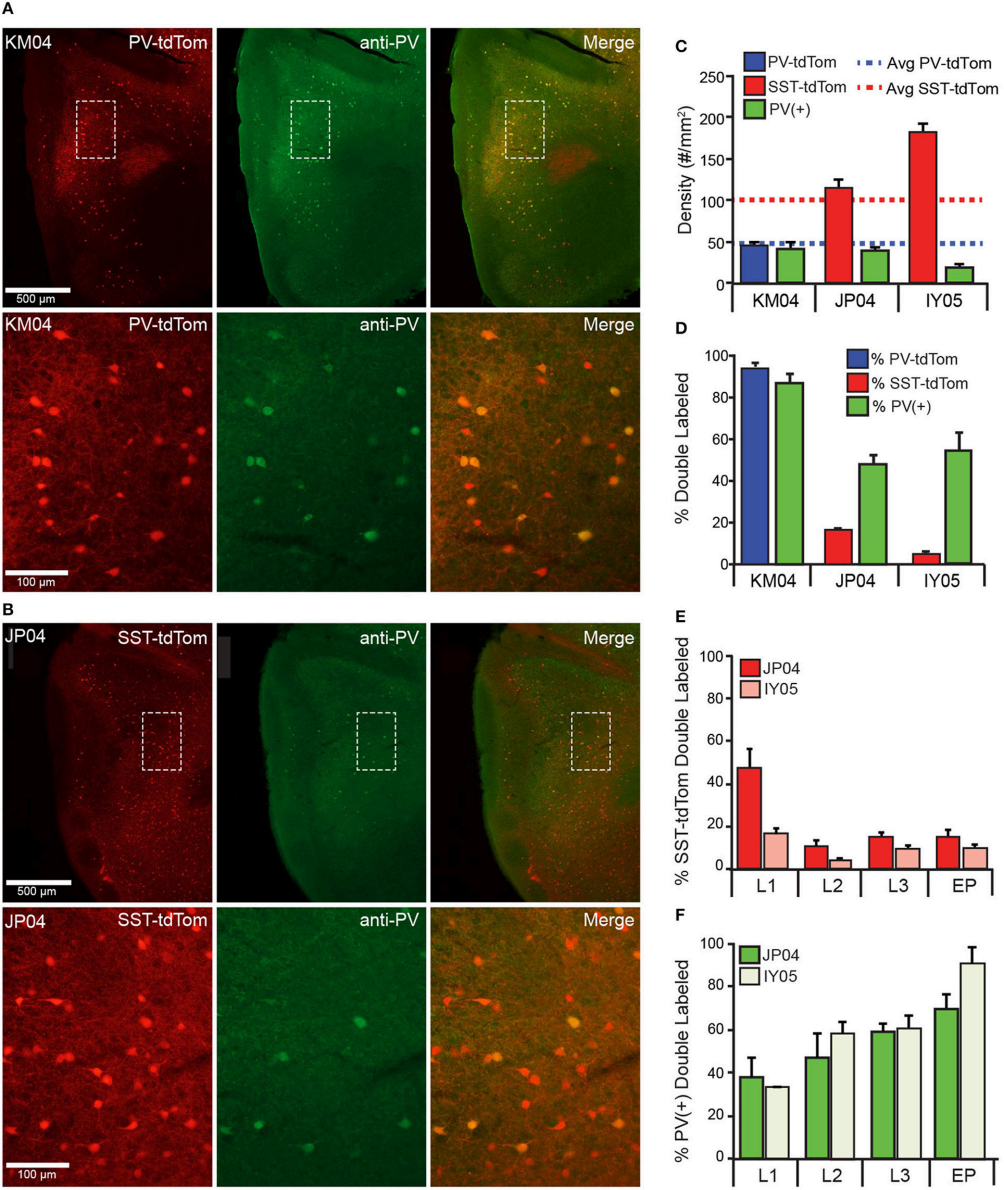
**Co-expression of parvalbumin and tdTomato in PV-Cre and SST-Cre lines**. Anti-parvalbumin immunostaining in **(A)** PV-tdTom mouse (KM04) and **(B)** SST-tdTom mouse (JP04). Sections from both animals were stained in the same experiment. Upper panels show the APC (4x magnification), lower panels show area indicated by dashed box at higher magnification (20x) Left panels: tdTom(+) neurons (red) in the PV-tdTom **(A)** or SST-tdTom **(B)** animals; middle panels: PV(+) interneurons labeled with GFP (green); and right panels: co-expression tdTom(+) and PV(+) cells (yellow). **(C)** Density of tdTom(+) cells in the PV-tdTom (KM04, blue) and two SST-tdTom (JP04, red; IY05, pink) mice. Dashed lines correspond to average densities of tdTom(+) cells reported for all SST-tdTom (red) and PV-tdTom (blue) mice. The green bars correspond to the density of PV(+) cells in each mouse. **(D)** Co-expression of PV(+) and tdTom(+) as a percentage of tdTom(+) cells in PV-tdTom (blue) or SST-tdTom (red) mice or as a percentage of PV(+) cells (green) in each mouse. **(E)** Co-expression of PV(+) and tdTom(+) as a percentage of tdTom(+) cells in SST-tdTom mice (JP04 red, IY05 pink) by layer. **(F)** Co-expression of PV(+) and tdTom(+) as a percentage of PV(+) cells in SST-tdTom tissue (JP04 dark green, IY05 light green) by layer.

Recent studies have suggested that the SST-cre transgenic line is vulnerable to off-target recombination and that ~6–14% of neurons express PV rather than SST (Hu et al., 2013; Pfeffer et al., 2013; Nassar et al., 2015). We analyzed anti-parvalbumin immunostaining [PV(+)] in tissue from one PV-tdTom mouse (KM04 Figure 4A) and two SST-tdTom mice (JP04, Figure 4A, and IY05) to quantify co-labeling between tdTom(+) and PV(+) cells in the two cre-lines. In KM04 and JP04, the average densities of PV(+) cells (42.1 ± 7.72, 39.5 ± 4.75 cells/mm^2^) were comparable to the average density of PV-tdTom cells reported above, but there were fewer PV(+) cells in IY05 (19.8 ± 3.08) (Figure 4C). As expected for the PV-cre line, 95 ± 3% of tdTom(+) cells were PV(+) in APC of PV-tdTom mice (KM04, Figures 4A,D). In the SST-tdTom animals, 6 ± 1% (IY05) and 16 ± 2% (JP04) of tdTom(+) cells were PV(+) (Figures 4B,D). Since a majority of recorded interneurons were in deep L2 and L3, we also investigated co-expression by layer in SST-cre mice (Figure 4E). Co-expression was variable in L1 (IY05: 18 ± 2%, JP04: 47 ± 9%) likely due to the low densities of SST and PV interneurons in this layer. The percentages of PV(+), SST-tdTom cells in L2 (IY05: 4 ± 1%, JP04: 10 ± 3%), L3 (IY05: 10 ± 1%, JP04: 15 ± 2%), and EP (IY05: 10 ± 2%, JP04: 15 ± 3%) were comparable to values reported for L4 of neocortex (Hu et al., 2013). Given the lower densities of PV cells in APC, a higher average proportion of PV(+) cells co-expressed tdTom in SST-cre animals (IY05: 57 ± 5%, JP04: 55 ± 4%) and co-expression varied across layers (Figure 4F). We also investigated whether PV-tdTom cells co-express SST using anti-somatostatin immunolabeling. We found 6 ± 1% of PV-tdTom cells were SST(+; Figure 1B4, *n* = 3 sections from KM04). Altogether, these findings suggest the potential for off-target recombination in PV-cells in the SST-cre line in APC is comparable to other cortical areas. In the following sections we will discuss variable degrees of influence off-target recombination may have on our results.

### Electrophysiological properties of SST and PV interneurons in piriform cortex

SST interneurons are commonly described as regular spiking (RS) or low-threshold spiking (LTS) while PV-interneurons are typically fast spiking (FS). However, a variety of spiking phenotypes, including FS, have been described for SST-cells (Ma et al., 2006; McGarry et al., 2010; Nassar et al., 2015). We endeavored to classify SST-cre cells in piriform cortex based on intrinsic subthreshold and suprathreshold electrophysiology. We used a hierarchical clustering algorithm to classify interneurons recorded in L3 from SST-cre, PV-cre, GIN, and G42 lines.

**Figure 5:**
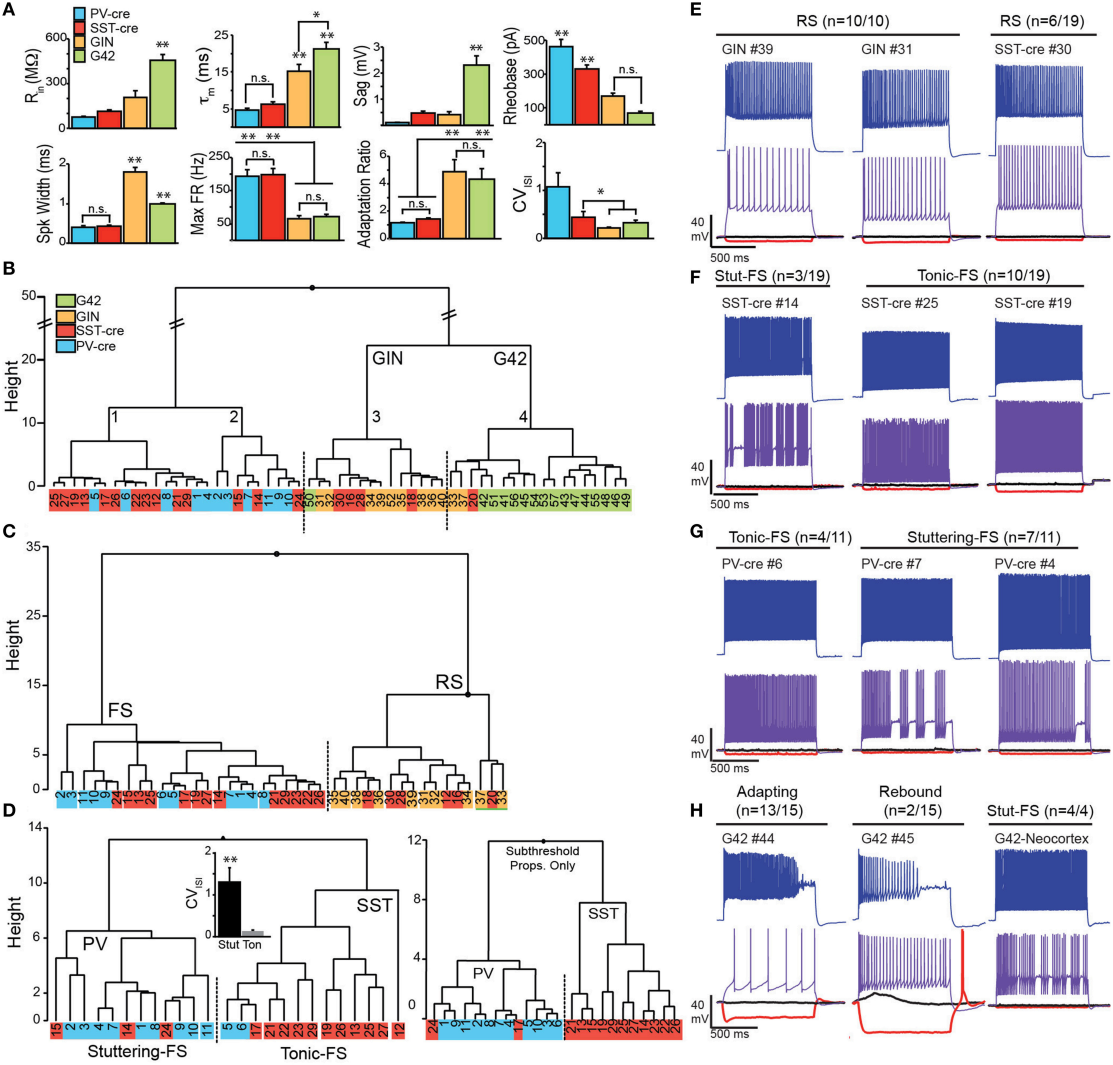
**Electrophysiological properties SST and PV cells in Cre, GIN, and G42 lines. (A)** Comparisons of subthreshold (*R*_in_, input resistance; τ_m_, time constant; Sag) and suprathreshold electrophysiological properties (Rheobase, spike width, maximum firing rate, adaptation ratio, and CV_ISI_: coefficient of variation of the interspike interval) in target neurons from anterior piriform cortex in SST-cre (red), PV-cre (blue), GIN (yellow), and G42 (green) mice. Statistical significance assessed using ANOVA-Tukey for multiple comparisons (^*^p < 0.05; ^**^p < 0.01, n.s., not significant). **(B)** Clustering of all interneurons using the eight electrophysiological parameters in **(A)**. **(C)** Clustering of SST-cre cells (red) as fast spiking (FS) with PV-cre (blue) or regular spiking (RS) with GIN (yellow) cells using eight parameters, in the absence of G42 cells. **(D)** In the absence of RS cells, FS cells cluster as either PV-cre or SST-cre with minimal error (five cells) using eight parameters (left). CV_ISI_(inset) significantly differs between stuttering FS-cells (mainly PV) and tonic FS cells (mainly SST; *p* < 0.01, unpaired *t*-test). SST-cre and PV-cre cells cluster with even less error (right, two cells) when only three subthreshold properties (*R*_in_, τ_m_, and Sag) are used. **(E)** Examples of RS SST cells in SST-cre and GIN mice. Numbers correspond to individual cells in clustering diagrams **(B–D)**. **(F)** Examples of FS SST-cre exhibiting both stuttering (Stut-FS) and tonic-FS responses. **(G)** Examples of FS PV-cre cells exhibiting both stuttering (Stut-FS) and tonic-FS responses. **(H)** Left, Examples of G42 cells recorded in APC showing unusual adapting and/or rebound responses. Right, Example of G42 cell recorded in neocortex showing classic stuttering-FS response.

Eight parameters were used for clustering—input resistance (*R*_in_), membrane time constant (τ_m_), sag, rheobase, spike width, max firing rate, adaptation ratio (AR), and coefficient of variation of the interspike interval (CV_ISI_; see Methods, Figure 5A). Initial statistical analysis of the intrinsic properties between the four cell classes (ANOVA-Tukey, Figure 5A) revealed that GIN and G42 cells differed from cells recorded in the cre-lines but did not distinguish between SST-cre and PV-cre cells. Following this, the interneurons were divided into two main branches and four clusters (Labeled 1–4, Figure 5B) using Ward's clustering algorithm. The first branch consisted of PV-cre cells and a majority of SST-cre interneurons while GIN and G42 neurons comprised the second branch (Figure 5B). SST-cre neurons showed the greatest diversity of responses and were found in all four clusters. G42 cells were the most homogeneous and all-but-one neuron comprised a single cluster (#4). Thus, we performed additional clustering analyses using neurons from the three clusters (#1–3) that encompassed the majority of SST-cre cells and excluded G42 cells. For each additional clustering analysis, *z*-scores were recalculated with respect to the members involved. In clustering analyses that included both the full data set (Figure 5B) and the three selected clusters (Figure 5C), a subset of SST-cre neurons consistently clustered with GIN cells. Like GIN cells, these neurons could be classified as RS (Figure 5E) and had intrinsic properties that, with the exception of spike width, did not significantly differ from GIN cells (Table 1). Since these neurons also significantly differed from the remaining SST-cre cells, we denoted these cells as RS SST-cre cells and removed them, along with GIN cells from additional clustering analyses. The electrophysiological responses of the remaining 13/19 SST-cre cells were indistinguishable “by eye” from those of PV-cre cells (Figures 5F,G). Both classes showed fast-spiking (FS) responses to depolarizing steps and had maximum firing rates near 200 Hz. However, direct statistical comparison of intrinsic properties between FS SST-cre cells and PV-cre cells revealed significant differences between these classes (Table 1). Further, cluster analysis based on the 8 parameters produced two clusters with minimal misclassification (*n* = 5/25, Figure 5D, left). Neurons clustered in the predominantly PV group (including SST-cre cells) were more likely to exhibit stuttering or irregular FS bursts characterized by a significantly greater CV_ISI_ (1.3 ± 0.3) than the SST group (0.13 ± 0.03, *p*: 0.002, *t*-test) which exhibit tonic-FS responses. Finally, we performed one final cluster analysis using just subthreshold parameters (*R*_in_, τ_m_ and sag) that are available under conditions when spiking is blocked. Surprisingly, three parameters were sufficient to produce two clusters corresponding to PV and SST cells, with just 2 misclassified SST-cre cells (Figure 5D, right). Sag was the factor that differed most significantly between SST-cre (0.48 ± 0.08 mV) and PV-cre (0.10 ± 0.02 mV, *p*: 0.0002, unpaired *t*-test) interneurons.

**Table 1:** **Intrinsic properties of identified interneurons in SST-Cre, PV-Cre, GIN, and G42 transgenic lines**.

**Cluster**	**Type**	***n***	***R*_in_**	**τ_m_**	**Sag**	**Rheo**	**SW**	**Max FR**	**AR**	**CV_ISI_**
RS	SST-cre	6	174 ± 47.5	12.7 ± 2.01	0.49 ± 0.16	200 ± 51.6	1.14 ± 0.19	72.0 ± 21.5	4.33 ± 1.03	0.56 ± 0.22
	GIN	10	206 ± 46.1	15.1 ± 2.13	0.41 ± 0.13	170 ± 21.3	**1.80** ± **0.13**	65.3 ± 6.96	4.86 ± 0.99	0.21 ± 0.03
FS	SST-cre	13	**113 ± 11.3**	**6.22 ± 0.59**	***0.48 ± 0.08***	**330 ± 28.6**	0.42 ± 0.02	199 ± 16.9	**1.44 ± 0.10**	0.43 ± 0.16
	PV-cre	11	74.0 ± 7.93	4.65 ± 0.39	0.10 ± 0.02	463 ± 40.9	0.40 ± 0.02	193 ± 19.5	1.17 ± 0.07	1.06 ± 0.39
G42	G42 APC	17	456 ± 39.1	21.3 ± 1.93	2.31 ± 0.36	70.6 ± 11.7	1.00 ± 0.05	71.8 ± 10.0	4.33 ± 0.93	0.31 ± 0.06
	G42 Neo	4	63.8 ± 3.83	4.25 ± 0.48	0.16 ± 0.12	566 ± 33.3	0.36 ± 0.07	224 ± 19.2	1.33 ± 0.15	0.46 ± 0.34

SST-cre interneurons were separated according to whether they clustered with regular spiking (RS) GIN cells or fast spiking (FS) PV-cre cells. Statistical comparisons of the mean ± SE were made between SST-cre cells and GIN cells in the RS cluster and between SST-cre cells and PV-cells in the FS cluster. Significant differences at a p < 0.05 level are indicated in bold and p < 0.01 are bold-italicized (unpaired t-tests). The mean ± SE values are also listed for G42 cells in anterior piriform cortex (APC) and G42 cells in neocortex but not statistically compared due to the low sample size of neocortical cells. R_in_, input resistance (MΩ); τ_m_, time constant (ms), Sag (mV); Rheo, rheobase (pA); SW, Spike width (ms); Max FR, maximum firing rate (Hz); AR, adaptation ratio; and CV_ISI_, coefficient of variation of interspike intervals.

Could misclassified SST-cre cells be due to off-target recombination in PV(+) cells? The percentage of misclassified SST-cre cells is ~10–16% (~2–3 cells of 19, Figure 5D). Given that we randomly targeted SST-tdTom cells in L3, the chance of selecting a PV(+) cell is expected to be ~6–16% (Figure 4D). Thus, it is possible that these “misclassified” cells may indeed be PV-cells.

To summarize, these findings demonstrate that SST neurons in piriform cortex can exhibit RS responses (*n* = 6/19) similar to GIN cells as well as FS-tonic (*n* = 10/19) and FS-stuttering (*n* = 3/19) responses like PV cells. Moreover, FS SST-cells are more frequently recorded (~70%) than RS cells (~30%) in piriform cortex. The similarity between FS SST cells and PV interneuron responses can make these classes difficult to distinguish in the absence of fluorescent markers. The prevalence of stuttering-FS responses in PV-cells over tonic-FS patterns in SST-cells suggests that firing pattern may be useful in this regard. However, given that SST-cells are far more likely to exhibit sag responses that are >0.25 mV, this criterion may be a better indicator of SST-cells.

For completeness we also evaluated the intrinsic properties of G42 cells in piriform/endopiriform areas. Intriguingly, despite the premise that these cells express PV, the intrinsic properties differed significantly from other cell classes in APC (PV-cre, SST-cre, GIN) as well as G42 cells recorded in neocortex of the same slices (Figure 5H). These G42 neurons are characterized by exceptionally high input resistance (*R*_in_: 456 ± 39 MΩ), time constant (τ_m_: 21 ± 2.0 ms), Sag (2.3 ± 0.4 mV), and spike frequency adaptation (AR: 4.3 ± 0.9; Figures 5A,H, Table 1). This suggests that G42 neurons form a cluster that is highly distinct from PV-cre cells in APC that are characterized by low *R*_in_ (75 ± 8.0 MΩ); τ_m_ (4.7 ± 0.4 ms); Sag (0.1 ± 0.02 mV); and AR (1.2 ± 0.07). Further, we did not record any PV-cre cells in L2/3 of APC with properties resembling G42 cells. These findings suggest that G42 cells represent a small, highly unique subgroup that are not representative of the majority of PV cells in APC. Thus, we abandoned crosses of G42 and SST-cre lines as a method to assess inhibition of PV-interneurons by SST-cells.

### SST-cells broadly inhibit L2/3 interneurons

To investigate inhibition mediated by SST-cells, we crossed SST-cre mice with Ai32 mice to express ChR2 and yellow fluorescent protein (YFP) in SST cells (SST-ChR2). We used brief flashes of blue light (see Methods) to evoke action potentials in SST-cells while recording IPSCs in postsynaptic, YFP(−) interneurons in lower L2 and L3. To investigate whether SST-mediated inhibition differs between interneuron subtypes in piriform cortex, we clustered YFP(−) interneurons based on the intrinsic properties available (*R*_in_, τ_m_, and Sag) in the presence of the sodium channel blocker, QX-314. We also included PV-cre cells (*n* = 9) recorded with QX-314 as benchmark neurons in the clustering analysis.

**Figure 6:**
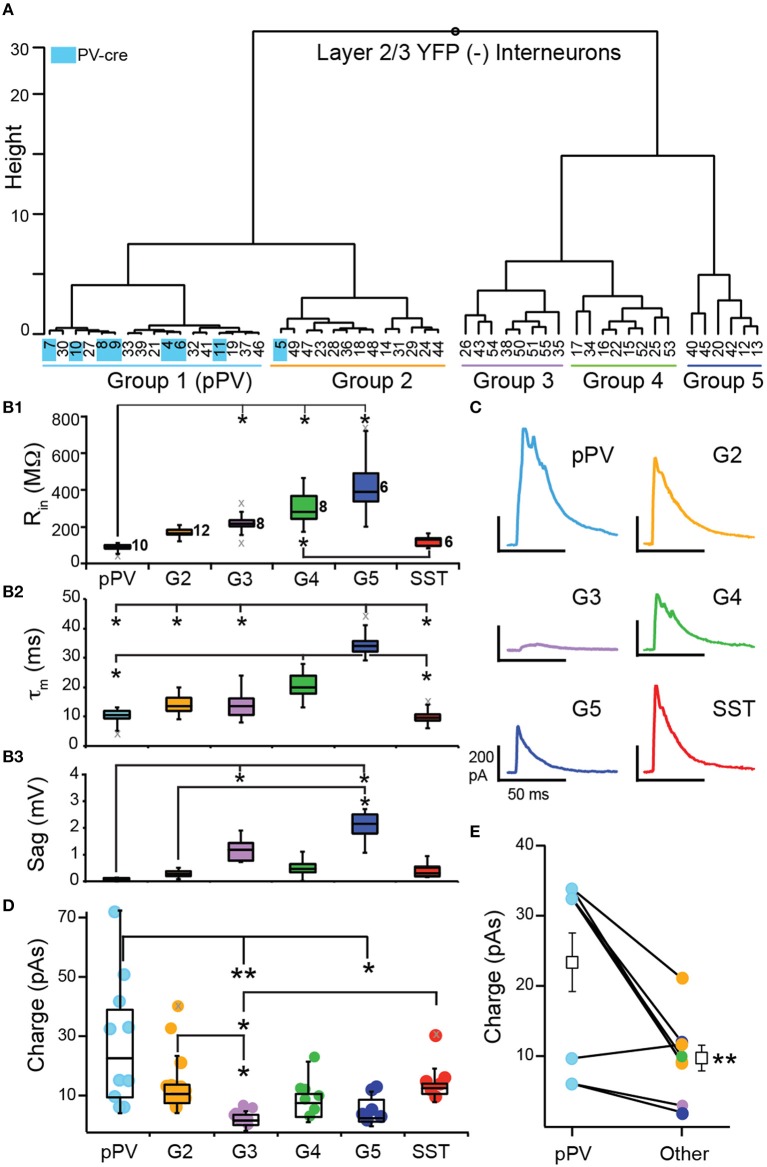
**Inhibition of L2/3 interneurons by SST-interneurons. (A)** YFP(−) interneurons were clustered according to subthreshold intrinsic properties into five groups (G1–G5). Identified PV-cre cells (indicated in blue) were included as benchmark neurons and clustered almost exclusively with G1. Thus, we denote G1 interneurons as pPV (blue). **(B)** Comparisons of subthreshold property distributions presented as median and quartiles (Q_1_, Q_3_) between groups (pPV, G2–G5, and SST; ^*^p < 0.05, KW-test) (**B1**) input resistance (*R*_*in*_), **(B2)** time constant (τ_m_), and **(B3)** Sag. Gray x's indicate outliers. **(C)** Representative IPSCs recorded in interneurons from each group. **(D)** Comparison of the distributions (median, Q_1_, Q_3_) of IPSC strength taken as the area under the IPSC (Charge, pAs) across groups (^*^p < 0.05, ^**^p < 0.01, KW-test). **(E)** Mean IPSC strength was significantly greater in pPV cells compared to other interneurons (G2–G5) recorded in the same slice (*p* < 0.01, WSR-test).

Clustering analysis produced two main branches. The first branch consisted of two clusters of interneurons with low *R*_in_ (< 200 MΩ) and Sag (< 0.30 mV) values [Group 1 (G1), G2; Table 2, Figures 6A,B]. Since nearly all of the benchmark PV-cre cells were found in G1, we putatively identified interneurons in this cluster as PV-cells (pPV). Moreover, G1 neurons differed significantly from G3 to G5 interneurons in many intrinsic properties (Figures 6B1–B3) suggestive of a distinct subclass. However, one PV-cre cell was found in G2 and the distributions of intrinsic properties of G2 did not significantly differ from G1 (*p* > 0.05, KW-test, Table 2). Thus, we cannot rule out the possibility G2 also contains some PV cells. The second branch had three possible clusters (G3, G4, G5) consisting of interneurons with higher *R*_in_ (>200 MΩ) and/or Sag (>1.0 mV) values than G1 or G2 (Table 2, Figures 5A,B). Although we cannot definitively identify these clusters as distinct subtypes of interneurons, Groups 3–5 appear to be distinguished by unique combinations of low or high *R*_in_, τ_m_, and Sag.

**Table 2:** **Intrinsic properties of interneurons that receive SST-mediated inhibition**.

**Cluster**	***n***	***R*_in_**	**τ_m_**	**Sag**	**IPSC (pAs)**
PV-cre	8	100 ± 13.7	7.38 ± 0.96	0.15 ± 0.03	n/a
G1 (pPV)	10	87.8 ± 6.52	9.90 ± 0.91	0.07 ± 0.02	28.2 ± 6.92
G2	12	166 ± 7.89	14.3 ± 1.01	0.28 ± 0.04	15.6 ± 3.16
G3	8	228 ± 20.2	22.5 ± 2.90	0.43 ± 0.18	12.7 ± 4.07
G4	8	218 ± 20.8	14.0 ± 1.81	1.18 ± 0.15	3.73 ± 0.80
G5	6	427 ± 75.4	34.8 ± 2.09	2.06 ± 0.25	6.39 ± 1.96
SST	6	120 ± 11.4	10.2 ± 1.08	0.56 ± 0.20	16.0 ± 2.97

The mean ± SE values for subthreshold intrinsic properties and IPSC strengths recorded in interneurons grouped according to the clusters shown Figure 5. Abbreviations and units as listed for Table 1.

Next we analyzed IPSC strength with respect to the groups of interneurons defined by cluster analysis (pPV, G2, G3, G4, G5) as well as identified SST-cells. Nearly all YFP(−) interneurons received inhibition (*n* = 43, 95%) from SST-cells. In addition, we recorded IPSCs in a small number of YFP(+) SST-ChR2 cells (*n* = 6/6). This latter finding was surprising because it has been previously reported that SST-cells do not inhibit each other in neocortex (Pfeffer et al., 2013). Representative IPSCs for each group are shown in Figure 6C. Since the IPSCs correspond to the inhibitory inputs from population of SST interneurons, strength was taken as the area (pAs) of the IPSC (Figure 6D, Table 2). IPSC strengths were highly variable in pPV cells (G1) but showed reduced variability in the other interneuron groups. The distributions of IPSC strengths differed significantly between pPV cells [median (M) 23.8; quartiles (Q_1_) 11.0; Q_3_ 39.8 pAs] and G3 (M 3.73; Q_1_ 2.66; Q_3_ 5.66 pAs, *p* < 0.01 KW-test) and G5 interneurons (M 4.66; Q_1_ 3.11; Q_3_ 10.6 pAs, *p* < 0.05 KW-test). IPSC distributions also differed between G3 and G2 (M 12.3; Q_1_ 9.10; Q_3_ 15.3 pAs, *p* < 0.05 KW-test) and SST interneurons (M 14.3; Q_1_ 12.4; Q_3_ 15.6 pAs, *p* < 0.05 KW-test). Finally, the mean inhibitory strength differed significantly between G2 (15.7 ± 3.2 pAs) and G3 (3.7 ± 0.8, *p*: 0.028, ANOVA-Welch), while the differences in means between G3 and pPV cells (28.3 ± 6.92 pAs, *p*: 0.051) and SST cells (16.4 ± 3.0, *p*: 0.053) were barely insignificant. These findings suggest that the mean and variability of inhibitory strength mediated by SST-cells may depend on the target interneuron.

We explored other explanations for correlations between interneuron groups and IPSC strength. We tested the possibility that IPSC strength was correlated with input resistance regardless of interneuron group (Supplemental Figure 1A). There was a weak, but significant, negative correlation (*R* = −0.36, *p*: 0.022, Pearson) between *R*_in_ and IPSC strength across all neurons. However, sequential removal of individual interneuron groups revealed this correlation was strongly biased by pPV cells that have the lowest *R*_in_ and the highest IPSCs values. In the absence of pPV cells, there is no correlation between *R*_in_ and IPSC strength (*r* = −0.29, *p*: 0.107). To investigate whether trends in the data may be attributed to animal or litter we plotted IPSC strength chronologically by animal and litter (Supplemental Figure 1B). We also plotted the mean strength (± 1 *SD*) for all IPSCs (14.3 ± 14.1 pAs). Altogether we recorded IPSCs in 49 interneurons in 28 slices from 18 animals from eight litters over the course of 1 year. Nearly all IPSCs regardless of animal or litter fell within 1 *SD* of the mean IPSC strength. Of the outliers, 5/7 were recorded in pPV cells and did not depend on litter or animal. Further, the mean IPSC strength calculated without the values for any one litter did not differ from the overall mean (Supplemental Figure 1C). Thus, there did not appear to be any significant trend in IPSC strength attributable to animal or litter.

All of our findings suggest that pPV cells are differentially inhibited by SST-cells. In 10/18 mice, multiple interneuron classes were recorded in a single animal. When pPV cells were one of the classes (*n* = 6/10 animals), pPV cells received the strongest inhibition in 5/6 cases. In the remaining 4 animals, G2 neurons, which are the most similar to pPV interneurons, received the strongest inhibition. Within slice comparisons revealed that pPV cells received significantly stronger inhibition (23.3 ± 4.17 pAs) than other classes (9.69 ± 1.84 pAs, *p*: 0.01, WSR-test; 4 slices, *n* = 8 comparisons, Figure 6E). While these findings are intriguing, they may be the most susceptible to off-target recombination in PV-cells, given that ~50% of PV(+) cells co-express tdTom in SST-tdTom mice (Figure 4F). Neocortical PV-cells can strongly inhibit each other but minimally inhibit other interneuron classes (Pfeffer et al., 2013). Although it is unlikely that the entirety of the inhibition received by pPV cells is mediated by PV-cell inhibition, it is possible that the additive effects of this inhibition could differentially contribute to the strength and variability of the population IPSCs. We attempted to quantify PV-to-PV cell inhibition but were unable evoke enough ChR2 current to elicit spike responses in PV-cells in slices from transgenic crosses (PV-cre × Ai32) or PV-ChR2 mice (Zhao et al., 2011). Thus, it would be important to verify these findings in the future using viral expression methodologies.

Nonetheless, we show that SST-cells provide inhibition to a majority of L3 interneurons and a variety of different interneuron classes in APC consistent with reports from other neocortical areas (Pfeffer et al., 2013; Jiang et al., 2015). Further, we find that SST-cells can inhibit each other in APC. These findings suggest that SST-cells play an important role in regulating network inhibition during olfactory processing in piriform cortex.

### Inhibition of pyramidal cells

**Figure 7:**
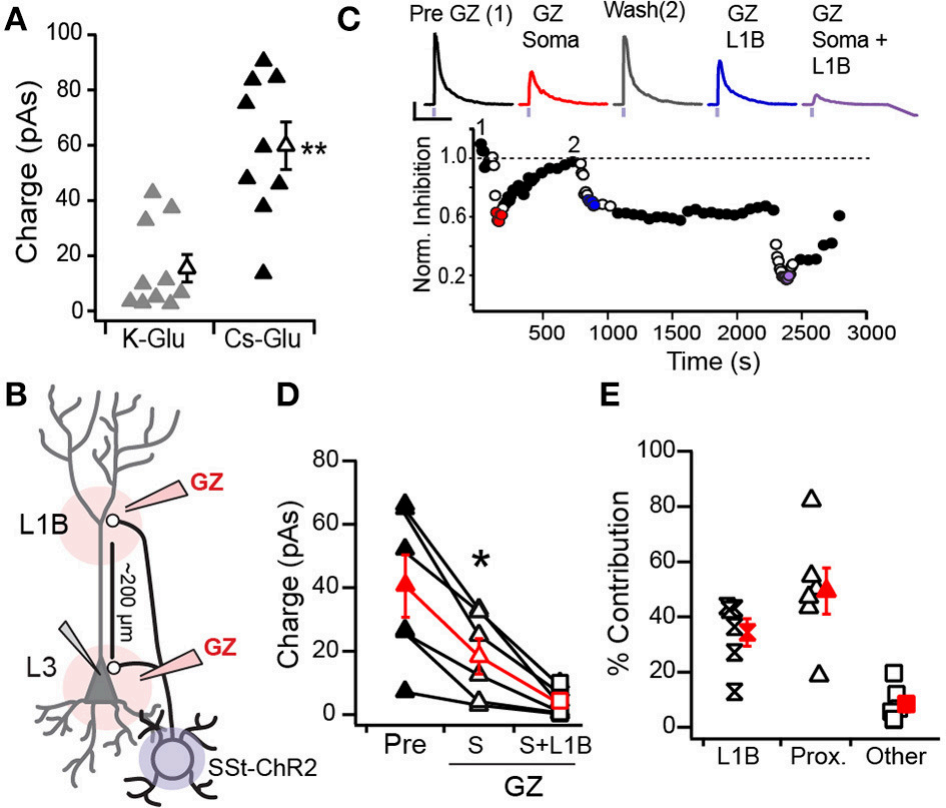
**Inhibition of L3 pyramidal cells by SST-interneurons. (A)** Mean IPSC strength significantly differed recorded in pyramidal cells with K-Gluconate (gray) vs. Cs-Gluconate (black) internal solutions (^**^p < 0.01, unpaired *t*-test). **(B)** Schematic of locations for distal (L1B) vs. proximal (L3, soma) gabazine (GZ) application. **(C)** Time course of IPSCs recorded in response to somatic and distal application of GZ in an example cell. IPSC strength was normalized to the average pre-GZ strength (black circles, 1). Open circles indicate GZ applications. IPSC responses to GZ application (top) correspond to average of colored circles (Red, GZ soma; Blue, GZ L1B; Purple, GZ L1B+Soma). IPSC amplitudes recovered between somatic and L1B applications (Wash, 2) but not following L1B application. **(D)** IPSC strength in response to somatic GZ application (open triangles) significantly differs from pre-GZ baseline (filled triangles) and somatic+L1B application (squares, ^*^p < 0.05, WSR-test). **(E)** The percent contribution of distal (L1B) and proximal (somatic) inhibition to the total inhibition recorded at baseline. “Other” corresponds to the percent of inhibition that remained during GZ application at both the soma and L1B.

Finally, we investigated the SST-mediated inhibition of L2/3 PCs in SST-ChR2 mice. All PCs recorded (*n* = 21) received inhibition from SST-cells. IPSCs were recorded at 0 mV using either K^+^-gluconate (*n* = 11) or Cs^+^-gluconate (*n* = 10) internal solutions. IPSCs were significantly stronger when using Cs^+^-gluconate (59.8 ± 8.59 pAs) than K^+^-gluconate (15.5 ± 4.98 pAs, *p*: 0.0003, unpaired *t*-test; Figure 7A). Since Cs^+^-gluconate solutions improve space clamp, we use this solution in the remaining experiments.

In a subset of L3 PCs (*n* = 6), we investigated SST-mediated inhibition of perisomatic regions vs. dendrites in L1B. Both regions have highly fluorescent neuropil in SST-tdTom mice indicative of projections from SST-cells (Figure 1). We locally applied the GABA_A_ receptor antagonist, Gabazine (GZ, 40 μM, see Methods) to the soma, to L1B, then to both sites simultaneously while recording light-evoked IPSCs in L3 PCs (schematic, Figure 7B). We chose L3 PCs because the somas and L1B were 221 ± 23.9 μm apart and offered better isolation of proximal vs. distal inhibition. Proximal inhibition was blocked by GZ application at the soma, while distal inhibition was blocked by GZ in L1B. Typically 10 min recovery time was allotted between applications at somatic and L1B sites. However, only two cells demonstrated sufficient recovery from GZ application at the soma to separately assess the contribution proximal inhibition by blockade of L1B (Figure 7C). None of the cells recorded showed sufficient recovery from L1B application in the time allotted (as seen in Figure 7C). Finally, GZ was simultaneously applied at both the soma and L1B. This blocked greater than 90% of the total inhibition in all cells.

Somatic GZ application significantly reduced inhibition from 40.6 ± 9.82 to 18.4 ± 5.59 pAs (*p*: 0.025 WSR-test, Figure 7D) suggesting that the remaining inhibition (~45%) is attributed to dendritic synapses. Simultaneous somatic and L1B GZ application further reduced IPSC strength (4.07 ± 1.64 pAs, *p*: 0.025, WSR-test). Since ~8 ± 3% of inhibition remained following simultaneous GZ application at both sites, we subtracted this value in our estimates of the percent contributions of somatic and dendritic inhibition. Overall, we find that distal L1B inhibitory synapses account for at least 39 ± 7% of the inhibition received by PCs while proximal somatic synapses provide 43 ± 7% (Figure 7E). These values did not significantly differ (*p* > 0.05, WRS-test). Thus, in addition to dendritic inhibition, our findings suggest that SST-cells also contribute to perisomatic inhibition of PCs. However, given that some PV-cells express ChR2 in SST-cre animals, further experiments are required to fully isolate the role of SST-cells in somatic inhibition. It is highly unlikely that off-target recombination in PV-cells contributes significantly to dendritic inhibition in L1B of PCs because PV-cells minimally project to this layer (Figures 1D, 2). Thus, these findings do confirm that SST-mediated inhibition regulates distal dendritic processing of afferent (L1A) and recurrent inputs (L1B) as predicted by anatomical projections.

## Discussion

The development of transgenic lines that selectively target SST-cells (SST-cre, GIN) or PV-cells (PV-cre and G42) has been highly beneficial with respect to understanding the roles of these interneurons classes in cortical processing. The target neurons in these lines have been described with respect to anatomical distribution, SST or PV expression, and intrinsic neuronal properties in a number of neocortical sensory areas including visual, somatosensory, and auditory cortex (Chattopadhyaya et al., 2004; Ma et al., 2006; Kuhlman and Huang, 2008; Oswald and Reyes, 2011; Hu et al., 2013). However, despite their utility, few studies have characterized these transgenic lines outside of neocortex (Oliva et al., 2000; Cabezas et al., 2013; Nassar et al., 2015). In this study we describe a number of important and novel findings with respect to the distributions, physiology, and connectivity of SST cells in piriform cortex.

### Distributions and electrophysiological properties of SST interneurons

We started with the simple assumption that the distributions and properties of interneurons selectively labeled in SST-cre, PV-cre, GIN, and G42 mice are comparable across sensory cortical areas regardless of paleocortical or neocortical location. The anatomical distributions of SST-cre and PV-cre cell somas and neuropil are consistent with immunohistochemical studies in olfactory cortex (Gavrilovici et al., 2010; Suzuki and Bekkers, 2010b; Kay and Brunjes, 2014). Specifically, the density of SST-cre cells is greater than PV-cre cells and the somas of both classes are predominantly located in deep layers of the APC. Further, SST-cre cells project to L1B and L2/3 while PV-cre cells project mainly to L2/3. However, SST and PV cells in the GIN and G42 lines, respectively, are sparse in piriform cortex. Although GIN and SST-cre cell populations overlap in APC, GIN cells account for only 2–6% of all SST-cells. G42 cells are located in deep L3 of APC and endopiriform cortex and have the strikingly different electrophysiological properties compared to PV-cre cells. Thus, while cre-lines encompass of the majority of SST and PV cells in piriform cortex, GIN and G42 lines only represent small subsets of SST or PV cell types. Thus, we caution against using GIN and G42 lines as the sole markers of SST or PV cells in piriform cortex.

In the absence of fluorescent markers, neocortical SST cells have been distinguished by broad spike widths and regular (RS) or low threshold (LTS) spiking patterns while PV cells are FS and have narrow spike widths (Kawaguchi and Kubota, 1996, 1997; Kubota and Kawaguchi, 2000; Nowak et al., 2003; Casale et al., 2015). These distinctions have been the basis for both *in vitro* and *in vivo* characterization of these classes. However, we find that ~70% of SST-cre cells in piriform cortex have FS responses and narrow spike widths that are superficially indistinguishable from PV-cre cells. Nonetheless, we show that SST and PV cells with FS properties can be differentiated with relatively low error using clustering algorithms and a number of suprathreshold (i.e., CV_ISI_) and/or subthreshold (i.e., Sag) properties.

At least three classes of SST-cells have been described based on selective GFP labeling in GIN, X98, and X94 transgenic lines (Ma et al., 2006; Nassar et al., 2015). Interneurons in the X94 line have FS and/or stuttering properties similar to PV cells (Ma et al., 2006; Xu et al., 2013). Neocortical X94 cells do not project to L1 but provide substantial inhibition to PV cells as well as excitatory cells located in the same or nearby layers (Ma et al., 2006; Xu et al., 2013). Our findings suggest that FS SST-cells in piriform cortex are similar to X94 cells and could locally inhibit PV and PCs in L2/3. Conversely, interneurons in the GIN and X98 lines exhibit RS responses and project axons to L1 (Ma et al., 2006). While the properties of RS cells in piriform cortex were more consistent with neocortical GIN cells than X98 cells, RS neurons were sampled more often (30%) than predicted by the sparseness of GIN cells (< 5% of SST cells) suggesting they may be akin to X98 cells. Nonetheless, the RS SST-cre cells we recorded are likely the same class of interneurons as SST(+), regular-spiking multipolar (rMP) cells that project to L1 of piriform cortex (Suzuki and Bekkers, 2010a). Taken together, our findings suggest at least two classes of SST cells exist in APC that are consistent with X94 and GIN/X98 classifications.

### SST-interneuron mediated inhibition

SST-cells are a major source of inhibition to PCs and a wide variety of interneurons in neocortex (Fino and Yuste, 2011; Pfeffer et al., 2013; Xu et al., 2013; Jiang et al., 2015). We find that SST-cells inhibited nearly all recorded neurons in APC, including PCs, SST cells, pPV cells, and four other unidentified types of inhibitory interneuron found in L2/3. With respect to PCs, we find that SST-cells strongly inhibit distal dendritic regions in L1B and likely, perisomatic regions. However, the latter could be contaminated in this study by off-target recombination in PV-cells. Nonetheless, our findings confirm a role for SST-cells in mediating dendritic inhibition in L1 and thus, regulating the flow of information from afferent (L1A) and recurrent (L1B) networks. An interesting possibility is that distal vs. proximal inhibition is mediated by RS (GIN/X98) vs. FS (X94-like) SST cells.

In contrast to previous findings that SST cells do not inhibit each other in neocortex (Pfeffer et al., 2013), we find that SST cells inhibit other SST cells in piriform cortex. Further, postsynaptic SST cells had subthreshold properties consistent with FS rather than RS SST-cells. This finding further supports the notion that different SST classes may play different functional roles in piriform cortex. We were unable to fully investigate the diversity or proportion of SST-interneurons inhibited because of ChR2 contamination. However, it is unlikely these findings are due to off-target expression in PV-cells. The rate of finding these cells was too high (6/6) given the low percentage (14%) of PV SST-tdTom cells that are PV(+). Moreover, PV cells rarely and only weakly inhibit SST-cells (Pfeffer et al., 2013).

Finally, SST cells inhibited the majority of interneurons recorded in L2/3 of piriform cortex as previously shown in neocortex (Pfeffer et al., 2013; Jiang et al., 2015). Although electrophysiological traits are not entirely indicative of neural class, we were able to cluster YFP(−) interneurons into five groups using the three subthreshold parameters (*R*_in_, τ_m_, and Sag). In particular, putative PV (pPV) interneurons clustered with identified PV-cre cells recorded under the same conditions. Interestingly, the distributions IPSC strengths co-varied with these interneuron groups. The strongest and most variable IPSC strengths were recorded pPV, Group 2 (G2), and SST interneurons. These groups were most similar with respect to *R*_n_, τ_m_, and Sag. Group 3 and 5 interneurons, tended toward higher *R*_n_, τ_m_, and Sag values, and received weaker, less variable inhibition. Group 3 interneurons in particular, routinely received weaker inhibition when compared with other interneuron types in the same slice or animal.

Since we did not know the interneuron groups in advance, multiple comparisons between groups were required, which greatly reduced overall statistical power. However, our findings suggest refined hypotheses are possible provided interneuron classes are identified. For example, when we used vasoactive intestinal peptide expressing interneurons (VIP-cre) as benchmark neurons (data not shown), these clustered with G3 interneurons. In other cortical areas, VIP interneurons preferentially target SST and PV cells over PCs (Lee et al., 2013; Pfeffer et al., 2013; Pi et al., 2013; Fu et al., 2014). Conversely, calbindin (CB) expressing interneurons have the highest density in APC and CB is frequently co-expressed in PV cells (Suzuki and Bekkers, 2010b). We speculate that G2 interneurons are CB-interneurons. A potential hypothesis is that SST cells provide stronger inhibition to PC-targeting interneurons (i.e., PV, CB, SST) vs. interneuron targeting interneurons (i.e., VIP cells). It is clear that additional and very different experiments are required to investigate these possibilities.

### Potential caveats

Expression patterns can differ between techniques that involve crossbreeding cre-mice with reporter or optogenetic lines like Ai14 or Ai32 vs. viral expression techniques. Using crossbreeding, we find consistent expression of tdTom as well as ChR2 evoked IPSC amplitudes across animals and litters. Further, there is more uniform spatial expression within animals. This is advantageous with respect to capturing weak or spatially dependent responses that would be susceptible to variable expression using viral techniques. However, a potential drawback of crossbreeding is off-target recombination in neurons that transiently express SST during development (Hu et al., 2013). We find that ~75–85% of SST-tdTom cells in APC express somatostatin [SST(+)] but this may be an underestimate given the variability of SST immunolabeling across cells. We also found that 6–15% of SST-tdTom cells express parvalbumin (PV(+)). It is unlikely that PV(+) labeling in SST-tdTom tissue is due to non-specific staining given the ~95% overlap of PV(+) and tdTom(+) cells in PV-tdTom animals. Finally, although interneurons that co-express PV and SST have been reported in L3 of rats (Cummings, 1997), we find that only 6% of PV-tdTom cells are SST(+). Thus, we cannot rule out off-target recombination as a factor in our experiments. Nonetheless, we expect that the our main findings are not qualitatively affected by off-target recombination since the majority of SST-cre cells are indeed SST(+) and only a small number are PV(+). However, we have highlighted results throughout that may be quantitatively susceptible. Going forward, it would be useful to verify the findings of this study using viral transfections in older animals that minimize the potential for developmentally regulated off-target recombination.

## Conclusions

In summary, we report several findings with respect to SST and PV interneurons in piriform cortex. First, GIN and G42 transgenic lines only represent a small subset of the SST and PV cell populations in piriform cortex. Second, only one-third of SST cells are classically RS and the remaining SST cells exhibit FS properties similar to PV cells suggesting at least two classes of SST-cells in piriform cortex. Third, SST cells inhibit distal dendritic and likely perisomatic regions of PCs. Fourth, SST cells provide broad inhibition to nearly all interneuron classes recorded, including SST and pPV cells. And fifth, SST cells may differentially inhibit interneuron classes such as PV cells. Our findings suggest that SST cells are poised to function in a number of different inhibitory circuits including dendritic regulation of afferent and recurrent excitation in PCs, inhibition of interneurons, and potentially disinhibition of PCs through SST-Interneuron-PC circuits. Overall, these findings suggest that SST-cells play a prominent role in regulating network excitation and inhibition during olfactory processing.

## Author contributions

AL and AO designed experiments. AL, NK, SM, AO collected data. AL, NK, AO analyzed data. AL and AO wrote manuscript.

## Funding

This study was funded by an NIH CRCNS grant (R01 DC015139) to AO.

### Conflict of interest statement

The authors declare that the research was conducted in the absence of any commercial or financial relationships that could be construed as a potential conflict of interest.

## References

[B1] Adesnik H. Bruns W. Taniguchi H. Huang Z. J. Scanziani M. (2012). A neural circuit for spatial summation in visual cortex. Nature 490, 226–231. 10.1038/nature11526 23060193 PMC3621107

[B2] Bekkers J. M. Suzuki N. (2013). Neurons and circuits for odor processing in the piriform cortex. Trends Neurosci. 36, 429–438. 10.1016/j.tins.2013.04.005 23648377

[B3] Cabezas C. Irinopoulou T. Cauli B. Poncer J. C. (2013). Molecular and functional characterization of GAD67-expressing, newborn granule cells in mouse dentate gyrus. Front. Neural Circuits 7:60. 10.3389/fncir.2013.00060 23565079 PMC3613764

[B4] Casale A. E. Foust A. J. Bal T. McCormick D. A. (2015). Cortical interneuron subtypes vary in their axonal action potential properties. J. Neurosci. 35, 15555–15567. 10.1523/JNEUROSCI.1467-13.2015 26609152 PMC4659822

[B5] Chattopadhyaya B. Di Cristo G. Higashiyama H. Knott G. W. Kuhlman S. J. Welker E.. (2004). Experience and activity-dependent maturation of perisomatic GABAergic innervation in primary visual cortex during a postnatal critical period. J. Neurosci. 24, 9598–9611. 10.1523/JNEUROSCI.1851-04.2004 15509747 PMC6730138

[B6] Chattopadhyaya B. Di Cristo G. Wu C. Z. Knott G. Kuhlman S. Fu Y.. (2007). GAD67-mediated GABA synthesis and signaling regulate inhibitory synaptic innervation in the visual cortex. Neuron 54, 889–903. 10.1016/j.neuron.2007.05.015 17582330 PMC2077924

[B7] Chen S. X. Kim A. N. Peters A. J. Komiyama T. (2015). Subtype-specific plasticity of inhibitory circuits in motor cortex during motor learning. Nat. Neurosci. 18, 1109–1115. 10.1038/nn.4049 26098758 PMC4519436

[B8] Chiu C. Q. Lur G. Morse T. M. Carnevale N. T. Ellis-Davies G. C. Higley M. J. (2013). Compartmentalization of GABAergic inhibition by dendritic spines. Science 340, 759–762. 10.1126/science.1234274 23661763 PMC3752161

[B9] Cottam J. C. Smith S. L. Häusser M. (2013). Target-specific effects of somatostatin-expressing interneurons on neocortical visual processing. J. Neurosci. 33, 19567–19578. 10.1523/JNEUROSCI.2624-13.2013 24336721 PMC3858626

[B10] Cummings S. L. (1997). Neuropeptide Y, somatostatin, and cholecystokinin of the anterior piriform cortex. Cell Tissue Res. 289, 39–51. 10.1007/s004410050850 9182599

[B11] Fino E. Yuste R. (2011). Dense inhibitory connectivity in neocortex. Neuron 69, 1188–1203. 10.1016/j.neuron.2011.02.025 21435562 PMC3086675

[B12] Fu Y. Tucciarone J. M. Espinosa J. S. Sheng N. Darcy D. P. Nicoll R. A.. (2014). A cortical circuit for gain control by behavioral state. Cell 156, 1139–1152. 10.1016/j.cell.2014.01.050 24630718 PMC4041382

[B13] Gavrilovici C. D'Alfonso S. Poulter M. O. (2010). Diverse interneuron populations have highly specific interconnectivity in the rat piriform cortex. J. Comp. Neurol. 518, 1570–1588. 10.1002/cne.22291 20187146

[B14] Haberly L. B. Price J. L. (1978). Association and commissural fiber systems of the olfactory cortex of the rat. II. Systems originating in the olfactory peduncle. J. Comp. Neurol. 181, 781–807. 10.1002/cne.901810407 690285

[B15] Hu H. Cavendish J. Z. Agmon A. (2013). Not all that glitters is gold: off-target recombination in the somatostatin-IRES-Cre mouse line labels a subset of fast-spiking interneurons. Front. Neural Circuits 7:195. 10.3389/fncir.2013.00195 24339803 PMC3857604

[B16] Jiang X. Shen S. Cadwell C. R. Berens P. Sinz F. Ecker A. S.. (2015). Principles of connectivity among morphologically defined cell types in adult neocortex. Science 350:aac9462. 10.1126/science.aac9462 26612957 PMC4809866

[B17] Kawaguchi Y. Kubota Y. (1996). Physiological and morphological identification of somatostatin- or vasoactive intestinal polypeptide-containing cells among GABAergic cell subtypes in rat frontal cortex. J. Neurosci. 16, 2701–2715. 8786446 10.1523/JNEUROSCI.16-08-02701.1996 PMC6578756

[B18] Kawaguchi Y. Kubota Y. (1997). GABAergic cell subtypes and their synaptic connections in rat frontal cortex. Cereb. Cortex 7, 476–486. 10.1093/cercor/7.6.476 9276173

[B19] Kay R. B. Brunjes P. C. (2014). Diversity among principal and GABAergic neurons of the anterior olfactory nucleus. Front. Cell. Neurosci. 8:111. 10.3389/fncel.2014.00111 24808826 PMC4010738

[B20] Kowianski P. Morys J. M. Wójcik S. Dziewiatkowski J. Luczynska A. Spodnik E.. (2004). Neuropeptide-containing neurons in the endopiriform region of the rat: morphology and colocalization with calcium-binding proteins and nitric oxide synthase. Brain Res. 996, 97–110. 10.1016/j.brainres.2003.10.020 14670636

[B21] Kubota Y. Kawaguchi Y. (2000). Dependence of GABAergic synaptic areas on the interneuron type and target size. J. Neurosci. 20, 375–386. 10627614 10.1523/JNEUROSCI.20-01-00375.2000 PMC6774130

[B22] Kuhlman S. J. Huang Z. J. (2008). High-resolution labeling and functional manipulation of specific neuron types in mouse brain by Cre-activated viral gene expression. PLoS ONE 3:e2005. 10.1371/journal.pone.0002005 18414675 PMC2289876

[B23] Lee S. Kruglikov I. Huang Z. J. Fishell G. Rudy B. (2013). A disinhibitory circuit mediates motor integration in the somatosensory cortex. Nat. Neurosci. 16, 1662–1670. 10.1038/nn.3544 24097044 PMC4100076

[B24] Ma Y. Hu H. Berrebi A. S. Mathers P. H. Agmon A. (2006). Distinct subtypes of somatostatin-containing neocortical interneurons revealed in transgenic mice. J. Neurosci. 26, 5069–5082. 10.1523/JNEUROSCI.0661-06.2006 16687498 PMC2020857

[B25] Madisen L. Mao T. Koch H. Zhuo J. M. Berenyi A. Fujisawa S.. (2012). A toolbox of Cre-dependent optogenetic transgenic mice for light-induced activation and silencing. Nat. Neurosci. 15, 793–802. 10.1038/nn.3078 22446880 PMC3337962

[B26] Marlin J. J. Carter A. G. (2014). GABA-A receptor inhibition of local calcium signaling in spines and dendrites. J. Neurosci. 34, 15898–15911. 10.1523/JNEUROSCI.0869-13.2014 25429132 PMC4244464

[B27] McGarry L. M. Packer A. M. Fino E. Nikolenko V. Sippy T. Yuste R. (2010). Quantitative classification of somatostatin-positive neocortical interneurons identifies three interneuron subtypes. Front. Neural Circuits 4:12. 10.3389/fncir.2010.00012 PMC2896209 20617186

[B28] Nassar M. Simonnet J. Lofredi R. Cohen I. Savary E. Yanagawa Y.. (2015). Diversity and overlap of parvalbumin and somatostatin expressing interneurons in mouse presubiculum. Front. Neural Circuits 9:20. 10.3389/fncir.2015.00020 26005406 PMC4424818

[B29] Nowak L. G. Azouz R. Sanchez-Vives M. V. Gray C. M. McCormick D. A. (2003). Electrophysiological classes of cat primary visual cortical neurons *in vivo* as revealed by quantitative analyses. J. Neurophysiol. 89, 1541–1566. 10.1152/jn.00580.2002 12626627

[B30] Oliva A. A. Jr. Jiang M. Lam T. Smith K. L. Swann J. W. (2000). Novel hippocampal interneuronal subtypes identified using transgenic mice that express green fluorescent protein in GABAergic interneurons. J. Neurosci. 20, 3354–3368. 10777798 10.1523/JNEUROSCI.20-09-03354.2000 PMC6773106

[B31] Oswald A. M. Reyes A. D. (2011). Development of inhibitory timescales in auditory cortex. Cereb. Cortex 21, 1351–1361. 10.1093/cercor/bhq214 21068186 PMC3097987

[B32] Pfeffer C. K. Xue M. He M. Huang Z. J. Scanziani M. (2013). Inhibition of inhibition in visual cortex: the logic of connections between molecularly distinct interneurons. Nat. Neurosci. 16, 1068–1076. 10.1038/nn.3446 23817549 PMC3729586

[B33] Pi H. J. Hangya B. Kvitsiani D. Sanders J. I. Huang Z. J. Kepecs A. (2013). Cortical interneurons that specialize in disinhibitory control. Nature 503, 521–524. 10.1038/nature12676 24097352 PMC4017628

[B34] Seybold B. A. Phillips E. A. Schreiner C. E. Hasenstaub A. R. (2015). Inhibitory actions unified by network integration. Neuron 87, 1181–1192. 10.1016/j.neuron.2015.09.013 26402602 PMC4635400

[B35] Stryker M. P. (2014). A neural circuit that controls cortical state, plasticity, and the gain of sensory responses in mouse. Cold Spring Harb. Symp. Quant. Biol. 79, 1–9. 10.1101/sqb.2014.79.024927 25948638 PMC4500789

[B36] Sturgill J. F. Isaacson J. S. (2015). Somatostatin cells regulate sensory response fidelity via subtractive inhibition in olfactory cortex. Nat. Neurosci. 18, 531–535. 10.1038/nn.3971 25751531 PMC4452122

[B37] Suzuki N. Bekkers J. M. (2010a). Distinctive classes of GABAergic interneurons provide layer-specific phasic inhibition in the anterior piriform cortex. Cereb. Cortex 20, 2971–2984. 10.1093/cercor/bhq046 20457693 PMC2978245

[B38] Suzuki N. Bekkers J. M. (2010b). Inhibitory neurons in the anterior piriform cortex of the mouse: classification using molecular markers. J. Comp. Neurol. 518, 1670–1687. 10.1002/cne.22295 20235162

[B39] Suzuki N. Bekkers J. M. (2012). Microcircuits mediating feedforward and feedback synaptic inhibition in the piriform cortex. J. Neurosci. 32, 919–931. 10.1523/JNEUROSCI.4112-11.2012 22262890 PMC6621151

[B40] Urban-Ciecko J. Fanselow E. E. Barth A. L. (2015). Neocortical somatostatin neurons reversibly silence excitatory transmission via GABAb receptors. Curr. Biol. 25, 722–731. 10.1016/j.cub.2015.01.035 25728691 PMC4393017

[B41] Wessa P. (2012). Hierarchical Clustering (v1.0.3) in Free Statistics Software (v1.1.23-r7). Office for Research Development and Education. Available online at: http://www.wessa.net/rwasp_hierarchicalclustering.wasp/.

[B42] Wilson D. A. Sullivan R. M. (2011). Cortical processing of odor objects. Neuron 72, 506–519. 10.1016/j.neuron.2011.10.027 22099455 PMC3223720

[B43] Wilson N. R. Runyan C. A. Wang F. L. Sur M. (2012). Division and subtraction by distinct cortical inhibitory networks *in vivo*. Nature 488, 343–348. 10.1038/nature11347 22878717 PMC3653570

[B44] Xu H. Jeong H. Y. Tremblay R. Rudy B. (2013). Neocortical somatostatin-expressing GABAergic interneurons disinhibit the thalamorecipient layer 4. Neuron 77, 155–167. 10.1016/j.neuron.2012.11.004 23312523 PMC3556168

[B45] Zhao S. Ting J. T. Atallah H. E. Qiu L. Tan J. Gloss B.. (2011). Cell type-specific channelrhodopsin-2 transgenic mice for optogenetic dissection of neural circuitry function. Nat. Methods 8, 745–752. 10.1038/nmeth.1668 21985008 PMC3191888

