# Supplementary material for: Inhibition by Somatostatin Interneurons in Olfactory Cortex
Source: Front Neural Circuits. 2016 Aug 17;10:62. doi: 10.3389/fncir.2016.00062 (PMC4987344; doi:10.3389/fncir.2016.00062)
Supplement: Supplementary file 1 [file Image1.PDF]

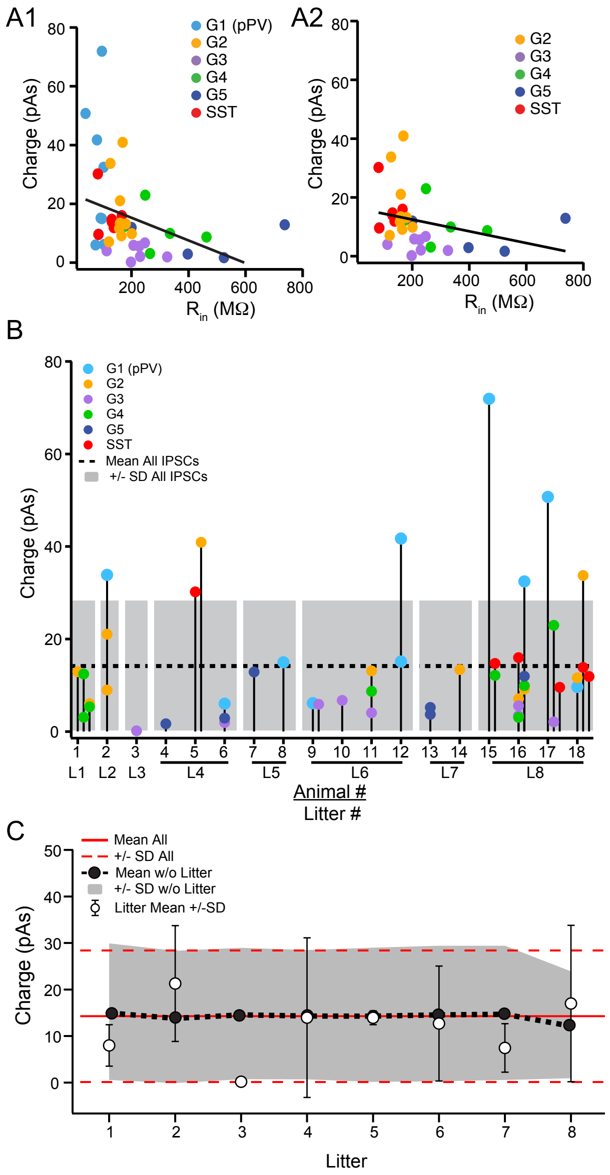


**Supplemental Figure 1: Other factors that could underlie differential inhibition by SST-interneurons.** A) IPSC strength is plotted against input resistance for each interneuron. The weak negative correlation is significant (R=-0.36, p: 0.022, Pearson) if pPV cells are included (A1) but not in their absence (A2, r=-0.29, p: 0.107). B) IPSC amplitudes are plotted with respect to interneuron group (colored circles, See Fig. 6 main text), for recordings in the same slice (vertical connecting lines) or animal (numbered) or litter (L). Also plotted is the mean IPSC strength (dashed horizontal line) +/- one SD (shaded). C) The mean IPSC strength +/-SD for each litter (white circles). Black circles and shaded area correspond to mean IPSC amplitude +/- SD calculated in the absence of the denoted litter. The overall mean +/- SD is also plotted (red lines). Removal of any one litter did not affect the overall mean or variance of the IPSCs.
